# Assessing Pupil Light Reflex Metrics in Glaucoma: Insights from a Systematic Review and Meta-Analysis

**DOI:** 10.1016/j.xops.2026.101225

**Published:** 2026-05-14

**Authors:** Mohammadreza Moniritilaki, Peter Wagner, Sieu K. Khuu

**Affiliations:** School of Optometry and Vision Science, University of New South Wales, Sydney, Australia

**Keywords:** Glaucoma, Pupillary light reflex, Diagnostic biomarkers, Systematic review, Meta-analysis

## Abstract

**Topic:**

This systematic review and meta-analysis aimed to identify the most affected pupil light reflex (PLR) metrics and determine the optimal stimulus conditions for detecting glaucomatous deficits.

**Clinical Relevance:**

The PLR is altered in glaucoma and understanding the basis of this change may provide a useful method for disease detection and progression monitoring.

**Methods:**

A comprehensive literature search was conducted across PubMed, Embase, and Google Scholar databases from June 2023 to April 2025. Studies evaluating PLR in glaucoma patients and healthy controls were included. Data extraction focused on PLR metrics such as amplitude of constriction, velocity of constriction and dilation, latency of constriction and dilation, and postillumination pupil response (PIPR). A meta-analysis was performed (Hedges' g) to determine the impact of glaucoma on each PLR metric. Subgroup and moderator analyses were conducted to assess the influence of dark adaptation, stimulus duration, and stimulus color on PLR metrics and to examine the relationship between PLR metrics and age, retinal nerve fiber layer (RNFL) thickness, and mean deviation (MD). This systematic review is registered with PROSPERO (CRD420251079002).

**Results:**

A meta-analysis revealed that the amplitude of constriction (the most affected metric), latency of constriction, velocity of constriction, and PIPR were significantly affected in mild glaucoma. Subgroup analysis suggested that the testing conditions involving a stimulus lasting >1 second, after dark adaptation of <5 minutes, are most sensitive for detecting PLR deficits. Moderator analysis indicated that age, RNFL thickness, and MD were associated with variations in PLR metrics. In severe glaucoma, the amplitude of constriction, duration of dilation, velocity of constriction, and PIPR showed the largest effect sizes (ESs). Overall, severe glaucoma led to larger ESs, indicating greater change in PLR metrics was associated with disease severity.

**Conclusions:**

These findings suggest that the amplitude of constriction is the most affected PLR metric in both mild and severe glaucoma. Furthermore, our results indicate that a stimulus lasting >1 second, when presented after a dark adaptation of <5 minutes, may represent the most sensitive condition for detecting amplitude of constriction deficits in mild glaucoma.

**Financial Disclosure(s):**

Proprietary or commercial disclosure may be found in the Footnotes and Disclosures at the end of this article.

Glaucoma is a progressive degenerative disease that leads to retinal ganglion cell (RGC) loss and optic nerve damage. Glaucoma is one of the leading causes of irreversible vision loss worldwide^1^ and is estimated to affect approximately 95 million people globally, with 10 million experiencing blindness in ≥1 eye, and many more enduring visual impairment that may significantly hinder their daily activities.^2^

Clinicians primarily rely on diagnostic techniques such as OCT and standard automated perimetry (SAP) to identify and monitor glaucoma progression.^3^ However, it is well recognized that these conventional methods have limitations in their diagnostic capabilities, particularly in early disease detection.^4^, ^5^, ^6^, ^7^ For example, SAP, in particular, is susceptible to substantial test–retest variability,^8^^,^^9^ a consequence of factors such as patient-related characteristics (including attention, fatigue, and comprehension of the task), fixation instability, false-positive and false-negative responses, and the inherently greater variability observed in regions with established visual field damage. This variability can obscure subtle functional deterioration, thereby reducing the sensitivity of SAP for identifying early progression.^10^ Additionally, the reliability of structural measurements using OCT can be influenced by variations in the surrounding anatomical structures and the presence of additional pathologies.^11^ These limitations can result in irreversible visual impairments, highlighting the importance of early detection. Consequently, research has increasingly focused on developing alternative or complementary diagnostic approaches.^12^^,^^13^

In addition to structural and functional deficits, glaucoma can also impair the pupil light reflex (PLR) as this disease affects retinal cells and neuronal connections underlying the PLR.^14^, ^15^, ^16^ The PLR refers to the pupil's protective reflex to light intensity, involving contraction (miosis) and subsequent dilation (mydriasis) of the pupil, which controls the amount of light entering the eye. A responsive pupil not only regulates the amount of light entering the eye but also improves the depth of focus and minimizes the visual impact of glare, diffraction, and optical defects.^17^ The PLR is a dynamic measure of autonomic nervous system activity and retinal function, characterized by a rapid pupil constriction to light, followed by a slower redilation after stimulus offset (Fig 1). After the onset of light, the dynamic pupil size change is initially characterized by the latency of constriction (which reflects the time between light onset and beginning of pupil constriction) and the amplitude of pupil constriction (typically defined as the difference between baseline pupil diameter [pupil size prior to light onset] and the minimum diameter of pupil during the reflex). Maximum and mean constriction velocity represent the fastest and average velocity of pupil constriction, respectively. Following light offset, the postillumination pupil response (PIPR) is observed, which includes the duration of dilation (i.e., the time required for the pupil to return to its baseline diameter) and mean dilation velocity (i.e., the speed/rate required to redilate back to baseline). Together, these components offer a comprehensive assessment of retinal and autonomic function, and alterations in these PLR components may signal underlying ocular or neurological disorders.

**Figure 1:**
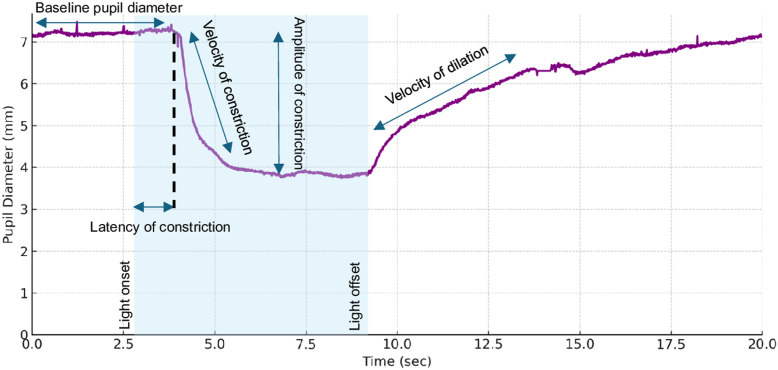
Time course of pupil diameter changes in response to the onset and offset of a bright light. After a brief delay (latency of constriction) from the onset of a bright light, the pupil quickly constricts (velocity of constriction), reaching a minimum pupil size (amplitude of constriction). After the offset of a bright light, the pupil gradually redilates (velocity of dilation), returning back to the baseline size.

It is well accepted that the PLR is driven by rod and cone photoreceptors, as well as intrinsically photosensitive RGCs (ipRGCs). Rods are thought to be involved in the PLR when triggered by low luminance of short-wavelength 470 nm (blue) light flashes. In contrast, cones are believed to respond to long-wavelength 646 nm (red) light flashes at moderate to high luminance levels. Additionally, ipRGCs are particularly sensitive to high luminance 470 nm continuous light.^16^^,^^18^^,^^19^ To analyze PLR changes due to disease, previous studies have quantified various PLR components in comparison with healthy controls (see Table 1), including amplitude of constriction, velocity of constriction and dilation, latency of constriction and dilation, and PIPR. Changes in ≥1 of these PLR components may indicate retinal dysfunction associated with disease processes. However, findings have been inconsistent regarding the degree of deficit, which may be related to differences in the methodologies used to measure the PLR.^19^, ^20^, ^21^, ^22^ Several studies have explored and quantified different PLR metrics and their change due to glaucoma using various methodologies, devices, and stimulus conditions.^20^^,^^22^, ^23^, ^24^ For example, in a study conducted by Feigl et al,^22^ a 10-second exposure to bright red and blue stimuli was used to measure the PIPR, which was defined as sustained pupil constriction for 30 seconds after stimulus offset. While significant changes in PIPR were observed in the severe glaucoma group when using a blue stimulus, the PIPR measured after a red stimulus was not affected.

**Table 1:** Definition of PLR Metrics (See Figure 1)

PLR Metrics	Definition
Amplitude of constriction	The maximum pupil constriction
Latency of constriction	Time between stimulus onset and beginning of pupil constriction
Duration of dilation	Time from peak constriction to redilated pupil size
Velocity of constriction	The average or maximum velocity of constriction
Latency of dilation	Time between stimulus off and beginning of dilation
Velocity of dilation	The average or maximum velocity of dilation
Postillumination pupil response (PIPR)	Pupil diameter after stimulus offset

PLR = pupil light reflex.

On the other hand, Gracitelli et al^18^ measured the PLR in glaucoma and control age-matched groups using 1 second blue and red flashes of 250 cd/m^2^ to stimulate inner and outer retina, respectively. Their results showed no difference in PIPR and amplitude of constriction between glaucoma and control groups for either color stimulus, indicating that the PLR was not affected by disease, or these stimuli were not sensitive enough to detect glaucomatous alterations. No correlation between SAP mean deviation (MD) and amplitude of constriction or PIPR was observed. Additionally, while no association between amplitude of constriction and retinal nerve fiber layer (RNFL) thickness was noted, a small correlation was found between RNFL thickness and PIPR after exposure to bright blue stimuli.

In another study, Najjar et al^14^ measured the PLR in patients with primary open-angle glaucoma and age-matched control using blue and red stimuli with gradually increasing the light intensity. Although no difference in pupillary constriction between groups was reported at lower irradiance level, pupillary constriction in patients with primary open-angle glaucoma progressively worsened as corneal irradiance increased. Additionally, the PLR deficit at high irradiance with blue light showed a stronger correlation with visual field loss and optic disc cupping, indicating that pupillary constriction in response to this stimulus may be more sensitive for detecting glaucomatous damage.^16^

Although evidence supports the presence of PLR deficits in glaucoma, findings have been inconsistent across previous studies, with some studies reporting contradictory results, highlighting the need for their systematic evaluation. This issue may stem from the fact that there is no standardized approach in testing, and previous studies have used various methodologies, stimuli, and devices to measure PLR, and have focused on quantifying different PLR metrics. A review of the literature shows many studies that have employed different stimuli with varying intensities, colors, durations, and sizes to target the different photosensitive cells, including rods, cones, and ipRGCs. Additionally, variations in pupillometry methods, dark adaptation duration, disease severities, and definitions of PLR metrics can also contribute to the differences in findings reported in the literature.

To address this gap, we conducted a systematic review and meta-analysis to identify the extent to which different PLR metrics are affected by glaucoma, as well as the optimal stimulus and methodology for detecting glaucomatous deficits. Specifically, we qualitatively reviewed recent studies that evaluated PLR changes in glaucoma and identified the various methods, approaches, stimuli, and measured different PLR metrics. A meta-analysis of studies that met our inclusion criteria was also conducted to determine the extent to which different PLR metrics are altered in glaucoma, with the goal of identifying which are the most affected, marking them as a possible biomarker for the disease. Additionally, subgroup analyses may identify the most sensitive stimulus conditions for detecting PLR defects associated with glaucoma, potentially leading to the identification of the optimal conditions for diagnosing glaucoma by quantifying PLR changes. Further moderator analyses examine the relationship between the PLR metrics and age, as well as structural (e.g., retinal nerve fiber density measurements) and functional loss (perimetric visual field MD and pattern standard deviation indices) in glaucoma. These analyses may be useful for estimating glaucomatous damage and monitoring disease progression, as both are routine clinical testing procedures.

## Methods

This systematic review was conducted according to the updated Preferred Reporting Items for Systematic Reviews and Meta-Analyses guidelines.^25^ This systematic review is registered with PROSPERO (CRD420251079002). As this study involved secondary analysis of previously published data and did not include any direct interaction with human participants or collection of identifiable information, institutional review board or ethics committee approval, informed consent, and compliance with the Declaration of Helsinki were not required. All included studies were assumed to have obtained appropriate ethical approval and informed consent as reported by their original authors.

### Search Strategy

A comprehensive literature review was conducted across the PubMed, Embase, and Google Scholar databases from June 2023 to April 2025, using the search strategy are detailed in Table S1, available at www.ophthalmologyscience.org. In addition, the literature search was performed from the reference list of selected research articles.

### Study Selection

Initial literature search was performed by 2 reviewers M.M. and S.K.K. in accordance with PICOS (Population, Intervention, Comparison, Outcomes, Study design) Principles.

a. Population: patients with glaucoma and a normal age-matched group.
b. Interventions: studies that evaluated PLR in glaucoma patients and control group were included.
c. Comparison: Studies that compared PLR between glaucoma and normal group were included.
d. Outcomes: outcome measures were PLR metrics as listed in Table 1.
e. Study design: cross-sectional and longitudinal studies.

**Figure 2:**
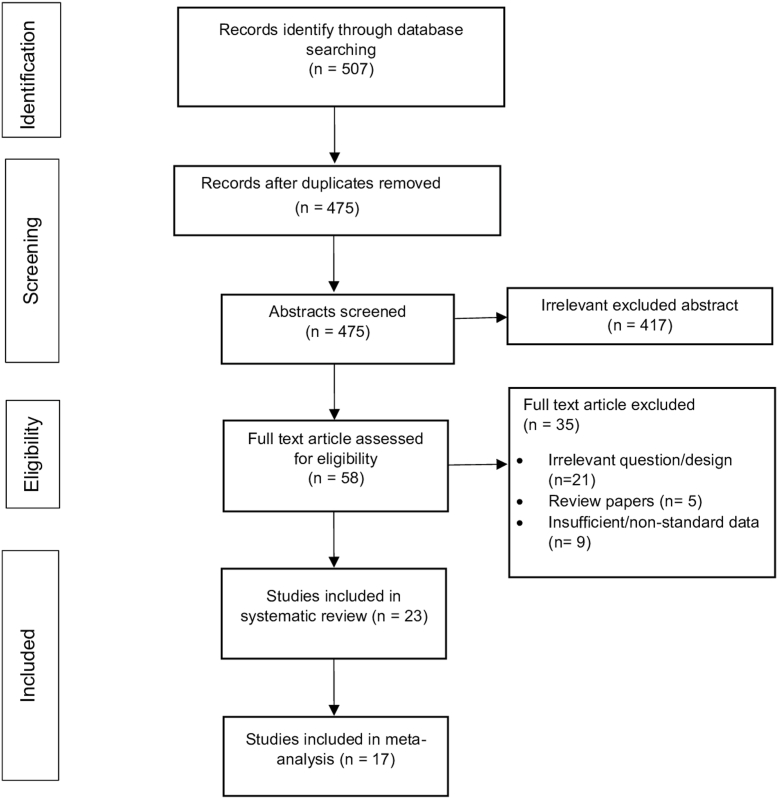
The outcomes of the study selection process outlining the number of studies initially identified, screened to meet the inclusion and exclusion criteria, and finally included in the study.

The process for selecting, retrieving, and filtering published studies followed the Preferred Reporting Items for Systematic Reviews and Meta-Analyses guidelines, as illustrated in Figure 2. From the studies identified in the search, only those that met the inclusion and exclusion criteria were included in the analysis (Table S2, available at www.ophthalmologyscience.org). These criteria were designed to include only studies that assessed the PLR in both glaucoma and normal groups, ensuring a reliable estimate of the effect size (ES) of PLR metrics, as testing conditions would be similar for both groups. Studies published prior to the year 2000 were excluded to ensure the review focused on research employing more modern PLR measurement technologies and standardized protocols. Importantly, methodologies capable of differentiating ipRGC-mediated responses only became available in the early 2000s; therefore, restricting inclusion to studies published from 2000 onward increased the relevance, consistency, and methodological robustness of the evidence synthesized in this review.

Studies that analyzed ≥1 of the PLR metrics listed in Table 1 for both glaucoma and normal groups were included. Studies that utilized overall PLR scores instead of individual PLR metrics, or those that examined differences between or within eyes, were excluded. Additionally, studies that did not specify the severity of glaucoma or did not provide participant demographic tables with visual field MD values to assess disease severity were also excluded. After the initial search, duplicate articles were eliminated. Titles and abstracts were then screened, and the full texts of eligible articles were independently reviewed based on the inclusion and exclusion criteria.

### Risk of Bias Assessment

The risk of bias for all included studies was evaluated using the Joanna Briggs Institute Critical Appraisal tools checklist, as shown in Table S3, available at www.ophthalmologyscience.org.^26^ Of the 23 studies, only 2 were rated as “unclear” or “no” on a few checklist items. This suggests that the overall risk of bias across the studies included in this systematic review and meta-analysis was low. Such low risk enhances the reliability and validity of the findings.

### Data Extraction

Glaucoma affects PLR metrics differently. While the amplitude of constriction, velocity of constriction and dilation, and PIPR have been reported to decrease due to glaucoma (negative ES), some parameters such as latency of constriction and duration of dilation have been reported to increase (positive ES), presumably due to reduction in processing speed.^22^^,^^23^^,^^27^ Target measures and relevant outcomes have been extracted from the included studies into a Microsoft Excel (Microsoft Corp) sheet. The extracted data included the study author, year of publication, number and age of participants, pupillometry details, stimulus color, duration, and intensity, all measured PLR metrics, RNFL thickness, MD, as well as the dark adaptation period in both groups. When studies presented data in formats other than mean and standard deviation, the data were converted to mean and standard deviation using appropriate formulas or procedures.

For studies that presented data graphically, data and variability were extracted using WebPlot Digitizer.^28^ The extracted data were used to perform a meta-analysis of studies that evaluate the PLR in glaucoma and age-matched healthy groups. The advantage of conducting a meta-analysis lies in its capacity to examine the combined impact of several studies, compensating for situations where a single study may not demonstrate any effect.

The alterations in PLR metrics for patients with glaucoma and age-matched normal participants were determined using Hedges’ g ES.^29^^,^^30^ Hedges' g was used as an ES metric to determine the impact of glaucoma on each PLR metric. Hedges’ *g* was calculated as the difference between the glaucoma and normal groups (glaucoma – normal). A positive ES indicates higher values in the glaucoma group, whereas a negative ES indicates lower values in the glaucoma group. An absolute ES value of <0.3 is considered small, between 0.3 and 0.5 is moderate, and >0.5 is regarded as a large difference between the compared values.^31^

In this study, Hedges' g was chosen instead of Cohen d because it takes the sample size into account, offering a more accurate estimate of the ES. To measure the impact of glaucoma on each PLR metric, Hedges' g ES was computed for each metric. This involved subtracting the mean of each specific metric in the healthy group from that in the glaucoma group and dividing the result by the pooled standard deviation, as per the following formula.

$${\text{Hedges}}^{'}g\;\text{ES}=g=\frac{M1—\;M2\;}{Pooled\;SD}$$

With M_1_ and M_2_ being the means of glaucoma and control group respectively. The pooled SD was calculated using the following formula.

$$\text{Pooled}\;\text{SD}=\sqrt{\frac{\left(n_{1}—1\right)SD_{1}^{2}+\left(n^{2}—1\right)SD_{2}^{2}}{n_{1}+n_{2}—2}}$$

Where *n*_1_ and n_2_ represent the sample sizes of the glaucoma and control groups, respectively, and SD_1_^2^ and SD_2_^2^ are the standard deviations of the glaucoma and control groups, respectively.

### Statistical Analysis

All statistical analyses and graph generation were conducted using Meta-Essentials V1.5^32^ and GraphPad Prism 9.5.1. To assess heterogeneity among studies in the meta-analysis, a random effects model was employed in both subgroup analysis and meta-regression. To evaluate the differences and variability in ESs within the meta-analysis, the Q statistic and I^2^ index were computed. The Q statistic indicates the statistical significance of heterogeneity,^32^ while the I^2^ index quantified the extent of heterogeneity. The I^2^ index was derived using the following formula.

$$I^{2}=\frac{Q–df}{Q}.100$$

$$Q=\sum_{i=1}^{k}w_{i}{\left(E_{i}–\bar{E}\right)}^{2}$$

With Q representing the heterogeneity value, df denoting the degrees of freedom, and k being the number of studies, W_i_ represents the weight for each study, E_i_ is the ES of the i^th^ study, and E¯ is the weighted mean ES. The I^2^ index, which ranges from 0 to 100, measured heterogeneity. A value of 25% indicates low heterogeneity, 50% indicates moderate heterogeneity, and ≥75% indicates high heterogeneity.^32^

The meta-analysis comprised of Hedges' g ES estimates, which were calculated for each individual PLR metric evaluated and pooled across studies that met the inclusion criteria. These ESs were then entered separately into the Meta-Essentials workbook^33^ for each metric and disease severity level. A random-effects model was applied to determine the combined ES for each PLR metric, reflecting the overall changes in these metrics for both mild and severe glaucoma groups. Subgroup analyses were also conducted using the same workbook to assess the impact of various stimulus factors, such as stimulus color and duration, as well as the duration of dark adaptation. *P* values from the analysis of variance in the subgroup analysis were used to identify any significant differences between subgroups. The influence of additional parameters such as age, MD, and RNFL thickness was examined through moderator analysis. Publication bias in the studies was evaluated using Egger test and visually represented with a Funnel Plot. The ESs were calculated separately for each single PLR metric across all included studies.

## Results

The selection process for studies included in our review is detailed in Figure 2, following the Preferred Reporting Items for Systematic Reviews and Meta-Analyses 2020 guidelines.^25^ Initially, 507 articles were retrieved and screened for duplicates. After removing 32 duplicate articles, 475 articles remained for abstract screening. During this stage, 417 articles were excluded for reasons such as not being written in English, being review articles, case series, retrospective studies, studies without a control group, or having irrelevant study outcomes. This left 58 articles for full-text review. After this review, 23 studies satisfied the inclusion criteria and were incorporated into the systematic review; 17 of the 23 studies had suitable data and contributed to the meta-analysis. Studies were omitted from the meta-analysis if their data could not be extracted or if they lacked an independent control group.

### Qualitative Analysis

Twenty-three studies underwent qualitative analysis (Table 2) that focused on study designs, pupillometry methods, and disease severities. It was noted that studies in this systematic review employed diverse methodologies to assess PLR in patients with glaucoma. Across these studies, a range of pupillometric techniques were used, with variations in stimulus types, colors, durations, and dark adaptation protocols. Additionally, a range of pupillometry techniques, including automated pupillometry, Ganzfeld stimulation system, Maxwellian view system, and custom-designed pupillometers equipped with infrared cameras were utilized to assess the PLR in both glaucoma patients and healthy individuals. The studies evaluated PLR alterations in both glaucoma patients and healthy control participants, aiming to enable early detection of glaucoma, assess disease progression, and investigate the correlation between pupillometric findings and structural and functional retinal changes.

**Table 2:** Summary of Key Participant and Pupillometric Methods and Testing Conditions of the 23 Studies Included in the Systematic Review

Study	Participants (Disease vs. Normal)	Age, Mean (SD) (Disease vs. Normal)	Pupillometry	Stimulus Color	Dark Adaptation	Stimulus Duration
Chang et al, 2013	148 vs. 71	67.4 (10.7) vs. 60.4 (9.6)	Automated pupillometry. RAPDx	White, red, green, blue, and yellow	1 min	Full field = 200 ms Patterned = 600 ms
Quan et al, 2023	46 vs. 23	41 (13.03) vs. 42 (11.08)	Custom-designed, HTC Vive Pro, with an integrated eye tracker	Blue	2 min	1 s
Ahmadi et al, 2020	15 vs. 17	70.7 (3.1) vs. 65.9 (3.2)	Automated pupillometry. DP2000 Binocular Pupillometer	Blue and red	5 min	20 s
Bayraktar et al, 2023	40 vs. 71	60.9 (9.6) vs. 57.6 (8.2)	Automated pupillometry. MonPack One, VisionMonitor System	White	5 min	200 ms
Feigl et al, 2011	25 vs. 16	60 (11.7) vs. 58 (12.9)	Maxwellian view optical system	Blue and red	Not mentioned	10 s
Duque-Chique et al, 2018	45 vs. 25	65.83 (1.2) vs. 54.27 (8.98)	Ganzfeld optical viewing system	Blue and red	10 min	1 s
Gracitelli et al, 2014	38 vs. 18	60.5 (11.2) vs. 56.2 (7.5)	Ganzfeld optical viewing system	Blue and red	10 min	1 s
Gracitelli et al, 2015	32 vs. 13	61.5 (11.6) vs. 56.8 (7.8)	Ganzfeld optical viewing system	Blue and red	10 min	1 s
Kankipati et al, 2010	16 vs. 19	63.7 vs. 59	Extended Maxwellian system equipped with LED light	Blue and red	Not mentioned	10 s
Kelbsch et al, 2016	25 vs. 16	61.8 (12.9) vs. 57.8 (13.1)	Mini Ganzfeld color LED stimulator	Blue and red	Not mentioned	1 and 4 s
Martucci et al, 2014	44 vs. 18	68.26 (5.46) vs. 63.2 (10.2)	Monocular automated dynamic pupillometry (MonCV3 Metrovision)	White	5 min	200 ms
Najjar et al, 2018	46 vs. 90	63.4 (8.3) vs. 61.4 (8.6)	Modified Ganzfeld viewing optical system	Blue and red	1 min	120 s of exponentially increasing light exposure
Najjar et al, 2021	149 vs. 173	68.5 (13.6) vs. 55.2 (26.7)	Custom-designed handheld pupillometer	Blue and red	0	9 s of exponentially increasing light exposure
Adhikari et al, 2016	46 vs. 21	67.8 (9.85) vs. 58.2 (9.2)	Maxwellian viewing optical system	Blue and red	10 min	1 s
Tatham et al, 2013	77 vs. 26	68.9 (12.3) vs. 50 (15.2)	Automated pupillometry. RAPDx	White	Not mentioned	200 ms
Tatham et al, 2014	66 vs. 50	69.1 (12.3) vs. 51.3 (15.2)	Automated pupillometry. RAPDx	White, red, green, yellow, and blue	Not mentioned	200 ms
Quan et al, 2023	74 vs. 23	63.4 (11.2) vs. 62.7 (10.8)	Custom-designed, HTC Vive Pro, with an integrated eye tracker	Blue and red	2 min	1 s
Wride et al, 2009	29 vs. 30	69.9 (13.6) vs. 59.6 (16.7)	Automated pupillometry. Pupilmetrix PLR60	White	Short	2 s
Nissen et al, 2014	11 vs. 11	65 vs. 62	Custom-designed pupillometer	Blue and red	1 min	20 s
Pradhan et al, 2017	59 vs. 0	60 (9.11) vs. 0	Automated pupillometry. RAPDx	White	2 min	200 ms
Pillai et al, 2019	130 vs. 43	56.9 vs. 35.21	Automated pupillometry. RAPiDo	White	Not mentioned	24 s
Chen et al, 2008	40 vs. 40	62.2 (9) vs. 52 (5.4)	Automated computer-based pupillometer	White	Not mentioned	1 s
Carle et al, 2015	19 vs. 24	64.1 (9.8) vs. 59.8 (7.3)	Automated multifocal pupillographic perimetry	Blue and yellow	Not mentioned	Blue = 750 ms Yellow = 330 ms

SD = standard deviation.

Eleven studies used automated pupillometry to measure PLR, with a particular emphasis on relative afferent pupillary defects.^20^^,^^21^^,^^34^, ^35^, ^36^ For example, Tatham et al^21^^,^^35^ employed the relative afferent pupillary defects pupillometer to record bilateral pupil responses to stimuli presented to 1 eye. These devices provide objective and computerized measurements of pupil reactions to light, offering greater precision and sensitivity than manual methods like the swinging flashlight test. The relative afferent pupillary defects pupillometer has proven especially effective in identifying asymmetric damage between the eyes in glaucoma patients,^21^^,^^35^ a key indicator of disease progression. Additionally, these devices use various colored stimuli to evaluate multiple parameters, such as pupil constriction amplitude, response latency, and PIPR.

Another widely used pupillometry technique is the Ganzfeld stimulation system and its modified versions, which allow researchers to present light flashes under controlled conditions. In our systematic review, 5 studies incorporated the Ganzfeld system or modified versions of it to present stimuli.^18^^,^^37^, ^38^, ^39^, ^40^ Gracitelli et al^18^ and Gracitelli and Paranhos^37^ both utilized a Ganzfeld stimulator to present blue and red chromatic stimuli for assessing the PLR in glaucoma patients. The Ganzfeld system was crucial for delivering uniform, full-field stimulation, which is important for evaluating overall retinal dysfunction. Additionally, 3 studies implemented the Maxwellian optical system to measure the PLR in glaucoma and healthy groups.^22^^,^^23^^,^^41^ This optical system provides consistent light exposure to the retina regardless of pupil size, ensuring all activated retinal area receive uniform illumination.^42^

Additionally, 4 other studies employed infrared cameras attached to custom-designed pupillometers to monitor pupil changes.^14^^,^^15^^,^^19^^,^^43^ For example, Quan et al^19^ utilized a Vive Pro virtual reality headset to present stimuli within a controlled environment designed to minimize variability. This method ensures accurate control of all experimental parameters, including timing, stimulus intensities, and dark adaptation. They incorporated 2 small eye trackers, employing Tobii technology, which were affixed to the HTC Vive Pro headset, enabling them to monitor pupil changes effectively.

Out of 23 studies, 16 utilized chromatic pupillometry to explore specific retinal pathways. Blue light stimuli were commonly employed to evaluate the activity of ipRGCs and rod photoreceptors. It is believed that a dim blue stimulus mainly targets rod cell function, while a bright blue stimulus is more effective in assessing ipRGC function, as demonstrated in the study by Feigl et al.^22^ Red stimuli are commonly used to assess cone photoreceptor function or serve as a reference for visualizing ipRGC activity. Additionally, only 7 studies employed white stimulation to measure pupil reaction (Table 2).^20^^,^^34^^,^^35^^,^^44^, ^45^, ^46^, ^47^

Of the 23 studies, 2 utilized distinct study designs to activate different retinal pathways.^14^^,^^39^ Najjar et al^14^^,^^39^ applied red and blue stimuli for 2 minutes and 9 seconds, respectively, with the intensity of the stimuli increasing over the duration of exposure. Because different retinal pathways (rods and cones) selectively respond to different light intensity ranges, increasing light intensity (within the same stimulus presentation) sequentially activated these pathways.^14^^,^^39^ The remaining 21 studies presented separate stimuli to target various retinal cells.

Among the 23 studies included, 7 utilized very short stimuli of <1 second.^20^^,^^21^^,^^34^^,^^35^^,^^45^^,^^48^^,^^49^ Another 7 studies employed a stimulus duration of 1 second to elicit the PLR.^15^^,^^18^^,^^19^^,^^23^^,^^37^^,^^40^^,^^46^ The remaining 9 studies used longer stimuli, ranging from 2 to 120 seconds.^14^^,^^22^^,^^36^^,^^38^^,^^39^^,^^41^^,^^43^^,^^44^^,^^47^

An additional variable across these studies was the dark adaptation period prior to stimulus presentation. Of the 23 studies, 4 implemented a 10-minute dark adaptation before stimulus presentation,^23^^,^^37^^,^^40^^,^^50^ while another 3 used a 5-minute period.^20^^,^^34^^,^^36^ Three studies employed a 2-minute dark adaptation^15^^,^^19^^,^^45^ and 3 others used a 1-minute period.^39^^,^^43^^,^^48^ The remaining 10 studies either did not specify the dark adaptation period,^21^^,^^22^^,^^35^^,^^38^^,^^41^^,^^44^^,^^49^ mentioned a short adaptation,^47^ or had no dark adaptation at all.^14^

Among the 23 studies reviewed, 12 classified glaucoma participants according to disease severity, while 11 did not. Of the 12 studies that did categorize participants, 9 used the Hodapp–Parrish–Anderson criteria, dividing them into mild, moderate, and severe categories based on visual field MD.^14^^,^^18^^,^^23^^,^^40^ Specifically, participants with an MD >–6 dB were considered to have mild glaucoma, those with an MD between –6 dB and –12 dB were classified as moderate, and those with an MD <–12 dB were categorized as having severe glaucoma. The remaining 3 studies employed different categorization methods. One study applied the Glaucoma Staging System 2, which classifies participants into 5 groups based on visual field measurement.^20^ Another study used the Monocular Esterman grid for categorization.^38^ Additionally, Feigl et al^22^ classified those having an MD >–6 dB classified as having mild glaucoma and those with an MD <–6 dB classified as having severe glaucoma.

### Quantitative Meta-Analysis

As mentioned, of the 23 articles included in the systematic review, 17 had sufficient data for a meta-analysis. The overall ES for each PLR metric was determined to identify its magnitude and to assess which metric(s) were most affected by glaucoma. Subsequently, subgroup analyses were conducted to investigate how certain stimulus conditions contributed to the observed ESs. In particular, subgroup analyses were conducted based on dark adaptation period (short: ≤5 minutes, long: >5 minutes), stimulus duration (short: ≤1 second, long: >1 second), and stimulus color (blue, red, and white). Importantly, these stimulus parameters are known to influence the PLR as changes in these variables target different retinal cells and or ipRGC neurons.

Moderator analyses were performed to quantify whether continuous variables, specifically visual field MD, RNFL thickness, and age, could explain variations in PLR ESs across studies. These factors were selected because MD and RNFL thickness are well-established indicators of glaucomatous damage,^51^ and aging is known to contribute to retinal cell loss.^52^ These analyses also aimed to determine whether these variables contributed to the heterogeneity observed between studies.

The aforementioned analyses were conducted separately for mild and severe disease studies. Participants with a MD <–12 dB were classified as having severe glaucoma, whereas those with an MD >–12 dB were considered to have mild glaucoma. For studies that did not explicitly categorize glaucoma severity, we applied the same classification criteria based on the MD values reported in the demographic tables.

### Publication Bias

To assess the presence of publication bias in the included studies, Egger regression test was conducted. The results indicated no significant evidence of publication bias (intercept = –0.45, 95% confidence interval: –2.04 to 1.15, *P* = 0.580). This suggests that small-study effects are unlikely to have influenced the overall findings of this meta-analysis. The symmetry observed in the funnel plot further supports the absence of substantial publication bias, reinforcing the robustness and reliability of the synthesized ESs (Fig S1, available at www.ophthalmologyscience.org).

### Mild Glaucoma

**Figure 3:**
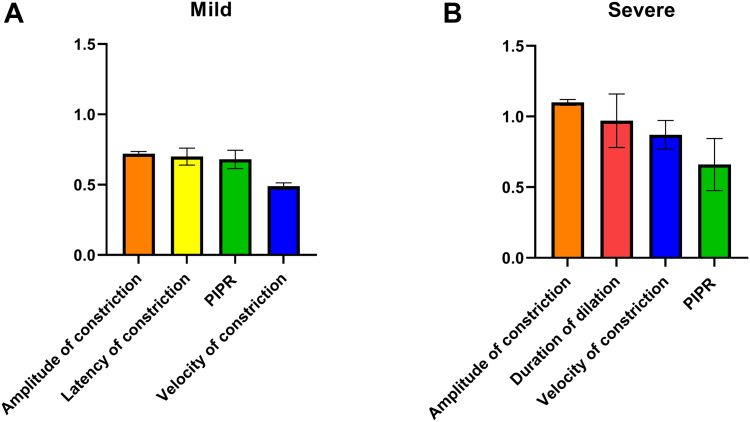
Absolute mean combined effect size values for different PLR metrics for mild (**A**) and severe glaucoma (**B**) analyses. Error bars represent 95% confidence intervals. PIPR = postillumination pupil response; PLR = pupil light reflex.

Our meta-analysis of mild glaucoma studies revealed that the amplitude of constriction, latency of constriction, PIPR, and velocity of constriction exhibited large ESs (Fig 3A). Our findings showed that amplitude of constriction in mild glaucoma (Fig S2, available at www.ophthalmologyscience.org) had a large ES (ES = –0.72, standard error [SE] = 0.06, Q = 267.92, *P* < 0.001, I^2^ = 80.96%). I^2^ indicated substantial heterogeneity among the included studies. To explore potential sources of the large heterogeneity and ES, subgroup analyses were conducted. These revealed that both stimulus duration (*P* < 0.0001) and dark adaptation duration (*P* = 0.002) significantly influenced the ES, with a long stimulus after a short dark adaptation associated with a larger ES. In contrast, stimulus color did not significantly affect the ES or heterogeneity (*P* = 0.58), suggesting that stimulus color does not contribute to the high heterogeneity and ES of the amplitude of constriction (Table 3). The observed high heterogeneity under these conditions may also be attributed to variations in study designs, stimulus type, and pupillometry methods. Furthermore, only MD (*r* = 0.07, *P* = 0.03) showed a significant but small positive correlation with the amplitude of constriction. A positive trend indicates that as these moderators increase, the ESs also increase, suggesting a reduction in glaucomatous damage. Moderator analyses indicated no statistically significant association between age and amplitude of constriction (*r* = –0.008, *P* = 0.40), nor between RNFL thickness and amplitude of constriction (*r* = 0.02, *P* = 0.07) (Table 4).

**Table 3:** Outcomes for Subgroup Analyses (Dark Adaptation Duration, Stimulus Duration, and Color) of Mild Glaucoma Studies

PLR metrics	Dark Adaptation		Stimulus Duration		Stimulus Color		
	Long (ES/p/I^2^)	Short (ES/p/I^2^)	Long (ES/p/I^2^)	Short (ES/p/I^2^)	Blue (ES/p/I^2^)	Red (ES/p/I^2^)	White (ES/p/I^2^)
Amplitude of constriction	–0.59 0.085 26.76%	–0.95 <0.001 87.86%	–1.26 <0.001 82.10%	–0.58 0.026 32.46%	–0.77 <0.001 84.36%	–0.69 <0.001 78.61%	–0.60 0.009 64.92%
Latency of constriction	0.8 0.165 31.63%	0.08 0.746 0.00%	-	-	0.88 0.5 0.00%	0.61 0.6 0.00%	0.61 <0.001 83.23%
Velocity of constriction	–0.63 0.999 0.00%	–0.32 0.357 9.1%	–0.22 0.188 42.38%	–0.52 0.2 21.34%	–0.48 0.38 7.29%	–0.44 0.05 57.87%	–0.54 0.09 47.37%
PIPR	–0.73 <0.001 73.19%	–0.64 <0.001 90.51%	–0.92 <0.001 84.52%	–0.54 <0.001 71.91%	–0.67 <0.001 88.17%	–0.78 0.08 51.54%	-

ES = effect size (Hedges’ g); p = *P* value; PIPR = postillumination pupil response; PLR = pupil light reflex.

**Table 4:** Outcomes of Moderator Analyses (RNFL Thickness, MD, and Age) for Different PLR Metrics in Mild Glaucoma

	Amplitude of Constriction		Latency of Constriction		PIPR		Velocity of Constriction	
	*r*	*P* Value	*r*	*P* Value	*r*	*P* Value	*r*	*P* Value
RNFL thickness	0.02	0.07	-	-	0.1	0.001	0.02	0.002
MD	0.07	0.03	–0.19	0.001	0.05	0.43	0.11	<0.001
Age	–0.008	0.4	0.1	0.01	–0.05	<0.001	–0.009	0.02

MD = mean deviation (visual field loss); PIPR = postillumination pupil response; PLR = pupil light reflex; *r* = correlation coefficient; RNFL = retinal nerve fiber layer.

The ES for the latency of constriction (Fig S3, available at www.ophthalmologyscience.org) was also large (ES = 0.70, SE = 0.09, Q = 31.11, *P* = 0.001, I^2^ = 67.85%). I^2^ indicated considerable heterogeneity across the included studies. Subgroup analyses suggested that stimuli presented after a long dark adaptation resulted in a larger ES, accompanied by lower heterogeneity (*P* < 0.001). Additionally, analysis based on stimulus color did not result in a statistically significant difference in ESs (*P* = 0.072), and due to insufficient data, the impact of stimulus duration on the ES and heterogeneity could not be evaluated (Table 3). Moreover, because of limited data, moderator analyses were limited to assessing the impact of MD and age on constriction latency (Table 4). A significant negative correlation between MD and latency of constriction (*r* = –0.19, *P* = 0.001) indicated that a more positive MD is associated with shorter constriction latency, suggesting less glaucomatous damage. Conversely, the significant positive correlation between age and constriction latency (*r* = 0.1, *P* = 0.01) suggests that older participants exhibit longer constriction latencies, possibly consistent with reported age-related RGC loss as noted by Harwerth et al.^52^

Our analysis also showed that PIPR (Fig S4, available at www.ophthalmologyscience.org) had a large ES (ES = –0.68, SE = 0.14, Q = 130.30, *P* < 0.001, I^2^ = 85.42%). The results of the subgroup analyses indicated that the ES was not influenced by dark adaptation duration (*P* = 0.73), stimulus duration (*P* = 0.143), or stimulus color (*P* = 0.657). The high heterogeneity across all subgroup conditions could be explained by variations in stimulus types and pupillometry methods. Additional studies are needed to further evaluate this metric. In addition, moderator analyses showed that unlike MD, which was not associated with PIPR (*r* = 0.05, *P* = 0.4), age and RNFL thickness were associated with the PIPR. A negative significant correlation between age and PIPR (*r* = 0.05, *P* < 0.001) indicates that with increasing age, ESs decrease (i.e., becomes more negative), indicating more deficit in PIPR. Conversely, a small positive significant association between RNFL thickness and PIPR (*r* = 0.1, *P* = 0.001) suggests that participants with thicker RNFL exhibit smaller (more positive) ES, indicating that PIPR may be less affected by disease in these individuals (Table 4).

The velocity of constriction (Fig S5, available at www.ophthalmologyscience.org) exhibited a medium ES (ES = –0.49, SE = 0.05, Q = 28.84, *P* = 0.069, I^2^ = 34.11%). The ES for constriction velocity was notably smaller compared to the other 3 PLR metrics. The subgroup analyses results indicated that the ES of constriction velocity under long dark adaptation was twice as large as that under short dark adaptation (*P* < 0.001), with significantly lower heterogeneity. Conversely, stimulus duration (*P* = 0.08) as well as stimulus color (*P* = 0.8) did not influence the ES (Table 3). However, the moderator analyses revealed a significant association between velocity of constriction and MD, RNFL thickness, and age (Table 4). Unlike age, which showed a negative association with velocity of constriction (*r* = –0.009, *P* = 0.02), the other 2 moderators showed positive associations, indicating larger MD (*r* = 0.11, *P* < 0.001) and RNFL thickness (*r* = 0.02, *P* = 0.002) are associated with larger (more positive) ESs, suggesting less glaucomatous damage. In contrast, increasing age is associated with slower constriction velocity.

### Severe Glaucoma

In studies evaluating severe glaucoma, the PLR metrics of amplitude of constriction, duration of dilation, velocity of constriction, and PIPR demonstrated large ESs (Fig 3B). Meta-analysis results demonstrated that the amplitude of constriction (Fig S6, available at www.ophthalmologyscience.org) showed a large ES (ES = –1.10, SE: 0.05, Q = 11.08, *P* = 0.982, I^2^ = 0.00%) with low heterogeneity, indicating consistent findings across studies. A total of 5 studies were included in this PLR metric quantitative analysis. Of the 24 ESs included, 15 were derived from a single study, which may explain the very low heterogeneity. Given this low heterogeneity, subgroup analyses were conducted. Neither dark adaptation duration (*P* = 0.422) nor stimulus color (*P* = 0.383) significantly influenced the ES of amplitude of constriction. Due to insufficient data, the effect of stimulus duration could not be evaluated (Table 5). The moderator analyses also reported no significant association between amplitude of constriction and age (*r* = - 0.002, *P* = 0.71), MD (*r* = 0.01, *P* = 0.41) and RNFL thickness (*r* = –0.01, *P* = 0.56) (Table 6).

**Table 5:** Outcomes for Subgroup Analyses (Dark Adaptation Duration, Stimulus Duration, and Color) of Severe Glaucoma Studies

PLR metrics	Dark Adaptation		Stimulus Duration		Stimulus Color		
	Long (ES/p/I^2^)	Short (ES/p/I^2^)	Long (ES/p/I^2^)	Short (ES/p/I^2^)	Blue (ES/p/I^2^)	Red (ES/p/I^2^)	White (ES/p/I^2^)
Amplitude of constriction	–1.15 0.98 0.00%	–1.03 0.5 0.00%	-	-	–1.07 0.88 0.00%	–1.19 0.97 0.00%	-
Duration of dilation	-	-	-	-	-	-	-
Velocity of constriction	-	-	-	-	-	-	-
PIPR	–1.57 <0.001 81.58%	–0.08 <0.001 85.20%	0.23 <0.001 85.75%	–1.10 <0.001 77.14%	-	-	-

ES = effect size (Hedges’ g); p = *P* value; PIPR = postillumination pupil response; PLR = pupil light reflex.

**Table 6:** Outcomes of Moderator Analyses (RNFL, MD, and Age) for Different PLR Metrics in Severe Glaucoma

	Amplitude of Constriction		Duration of Dilation		Velocity of Constriction		PIPR	
	*r*	*P* Value	*r*	*P* Value	*r*	*P* Value	*r*	*P* Value
RNFL thickness	–0.01	0.56	-	-	–0.26	0.27	-	-
MD	0.01	0.41	-	-	0.03	0.04	–0.08	0.3
Age	–0.002	0.71	–0.1	0.26	–0.003	0.8	–0.04	0.09

MD = mean deviation (visual field loss); PIPR = postillumination pupil response; PLR = pupil light reflex; *r* = correlation coefficient; RNFL = retinal nerve fiber layer.

The ES for duration of dilation (Fig S7, available at www.ophthalmologyscience.org) was significant and large (ES = 0.97, SE = 0.18, Q = 19.64, *P* = 0.001, I^2^ = 74.55%). I^2^ indicates substantial heterogeneity among included studies. However, due to insufficient data, subgroup analyses were not feasible (Table 5). Additionally, due to lack of data, the association between age and duration of dilation was only examined. This analysis reported no significant relationship between these variables (*r* = –0.1, *P* = 0.26), indicating the high heterogeneity is not associated with the age-related variations among the participants (Table 6).

The velocity of constriction (Fig S8, available at www.ophthalmologyscience.org) was another PLR metric with a large ES (ES = –0.87, SE = 0.11, Q = 8.44, *P* = 0.208, I^2^ = 28.88%). I^2^ indicated low heterogeneity in the ESs among the evaluated studies. Of the 7 included ESs, 6 were derived from the same research group, which may explain the low heterogeneity. Due to insufficient data, subgroup analyses were not performed (Table 5). However, moderator analyses showed that MD was the only moderator significantly associated with the velocity of constriction (*r* = 0.03, *P* = 0.04) (Table 6).

Our results also reported that the PIPR (Fig S9, available at www.ophthalmologyscience.org) is greatly affected (as indicated by a large ES) in severe glaucoma (ES = –0.66, SE: 0.32, Q = 95.22, *P* < 0.001, I^2^ = 86.35%). Subgroup analyses indicated that both stimulus duration (*P* = 0.007) and dark adaptation duration (*P* = 0.002) influenced the ES, with a short stimulus after a long dark adaptation associated with a larger ES. However, the evaluation of the effect of stimulus color on the overall ES was not possible due to insufficient data (Table 5). Further moderator analyses showed no significant effect of age (*r* = –0.04, *P* = 0.09) or MD (*r* = –0.08, *P* = 0.3) on PIPR. There was insufficient data to assess the effect of RNFL thickness (Table 6). The high heterogeneity observed across the studies could be attributed to differences in study designs, stimulus characteristics, and pupillometric devices.

## Discussion

Our study finds the amplitude of constriction is the most affected metric in both mild and severe glaucoma groups, implicating that this PLR metric may be most sensitive to glaucomatous related retinal changes. As expected, the ES for amplitude of constriction was larger in the severe group (with more retinal damage) than in the mild group (Fig 3). Subgroup analyses further revealed that the largest ESs were observed when a stimulus lasting >1 second was used after a dark adaptation period of up to 5 minutes.

Overall, our findings suggested that PLR is influenced by glaucoma. However, the results of our meta-analysis indicated that glaucoma affects PLR metrics to different degrees. Some metrics exhibited more significant deficits, while others showed fewer alterations (Table S4, available at www.ophthalmologyscience.org).

These findings align with conclusions from other review studies;^27^^,^^53^ however, the present study provides a comparative and quantitative analysis of how individual PLR metrics are affected by glaucoma.

As mentioned, our results indicated that the amplitude of constriction is the most affected by glaucoma (Fig 3). It is well established that the PLR is driven by retinal photoreceptors^24^ as well as ipRGC.^54^^,^^55^ While glaucoma predominantly affects RGCs,^56^ there is evidence suggesting glaucoma-related photoreceptor alteration also occurs.^57^ Therefore, the reduced amplitude of constriction observed in glaucoma patients can be attributed to diminished input from photoreceptors and ipRGCs to the olivary pretectal nucleus, a critical structure involved in mediating the PLR. This finding is supported by the results of our subgroup analyses of amplitude of constriction in mild glaucoma, which demonstrated a higher ES with long stimulus duration compared with a short stimulus duration (Table 3). This is consistent with and reflects the response of ipRGC cells that are slower and more sustained than photoreceptor-driven responses.^58^, ^59^, ^60^ Our meta-analysis confirms and quantifies the reduction in amplitude of constriction previously observed in individual studies,^15^^,^^18^^,^^19^^,^^23^^,^^48^ providing pooled ES estimates, highlighting its potential as a biomarker. Although ESs were larger in severe glaucoma, the low heterogeneity observed in this subgroup likely reflects methodological consistency among the included studies, which is due to the smaller number of studies using similar stimulus protocols, rather than indicating greater reliability of the ES. Further research should standardize stimulus conditions to allow clearer interpretation of disease-dependent effects.

The latency of pupil constriction was also affected in the mild glaucoma group (Fig 3A). Particularly, individuals with glaucoma exhibit a longer latency of constriction compared with the normal group, which may be a sign of photoreceptor damage and reduced photoreceptor density in glaucoma patients.^57^^,^^61^ While our meta-analysis demonstrated an overall significant delay in constriction latency, individual studies varied in their findings. For example, Chang et al^48^ reported longer constriction latency in glaucoma patients, whereas Bayraktar et al^34^ did not observe significant differences between glaucoma and healthy controls. This discrepancy may stem from factors such as the smaller sample size in the Bayraktar et al’s study (40 participants) compared with the study by Chang et al (148 participants), which also had on average older glaucoma patients (67 years vs. 60.9 years), or variations in the automated pupillometry techniques employed in each study. Highlighting these differences helps explain the heterogeneity observed in our pooled analysis.

The duration of dilation was also another PLR metric affected by severe glaucoma (Fig 3B). Despite reduced amplitude of constriction^20^^,^^39^^,^^48^ and significant damage to ipRGC,^22^ which would typically be expected to result in a shorter duration of dilation, our study found the opposite effect of a prolonged duration of dilation. This can be explained by the possibility that the remaining ipRGC compensate for retinal cell loss and contribute to a sustained pupil response, thereby leading to prolonged dilation. Similar findings have been reported by Quan et al,^15^^,^^19^ who observed longer dilation duration in both mild and severe glaucoma groups. They also found a negative correlation between pupil dilation time and structural retinal parameters. They reported that prolonged dilation time was associated with thinning of the inner retinal layers. These observations suggest that structural retinal damage, particularly in the macular region, may impair neurotransmission and slow signal processing, contributing to delays in the pupil returning to its baseline size.

The velocity of constriction was affected in both mild and severe glaucoma groups (Fig 3). The negative ES indicated that velocity of constriction is higher in the normal group relative to the glaucoma group. The reduced constriction velocity is attributed to damaged photoreceptors and RGCs as well as thinning of retinal structures,^19^^,^^20^^,^^39^^,^^48^ which collectively result in delayed signal transmission and slower pupil constriction. Additionally, our findings showed that ESs were dependent on the dark adaptation duration in mild glaucoma (Table 3). Particularly, studies employing longer periods of dark adaptation demonstrated a larger ES, which indicates a significantly slower constriction velocity compared with studies with shorter dark adaptation. This may be attributed to the dark adaptation characteristics of photosensitive retinal cells. Cone cells regain their sensitivity quicker, within 5–10 minutes, whereas rods require a longer duration to recover.^62^ Consequently, in studies with shorter dark adaptation, the response to stimuli primarily involves cone cells. Hence, the slower constriction velocity observed in such cases likely reflects cone photoreceptor cell loss associated with glaucoma. Conversely, in studies with longer dark adaptation, both cone cells (which are fully dark-adapted) and partially recovered rods contribute to the pupil response. This broader cellular involvement in pupil response results in larger ES, reflecting damage to a wider range of retinal cells. Therefore, studies with longer dark adaptation exhibit a larger ES for constriction velocity.

The PIPR after a light stimulus is a PLR metric that is affected in both mild and severe glaucoma groups (Fig 3). Similar to the velocity of constriction, the negative ES reflects a shorter PIPR in glaucoma patients, which is likely due to greater ipRGC damage. It is well established that the sustained pupillary response after stimulus offset is primarily driven by ipRGC functionality.^24^ Given that glaucoma primarily affects RGCs,^56^ the impaired function of ipRGCs may explain the reduced sustained pupillary response observed in these patients. Consequently, the PIPR can be considered as a highly sensitive biomarker for glaucoma detection. Further research is needed to elucidate its diagnostic utility and underlying mechanisms.

After establishing that the amplitude of constriction is the most impacted PLR metric in glaucoma, further subgroup analyses revealed that the ES of amplitude of constriction in mild glaucoma after short dark adaptation (up to 5 minutes) is larger than that observed with long dark adaptation, indicating that shorter dark adaptation conditions may be more sensitive in detecting glaucomatous changes in the PLR (Table 3). Shorter dark adaptation likely captures cone-dominated responses, which may be more affected in early-stage glaucoma. This is in line with the studies that showed S-cone pathway exhibits significant damage in early primary open-angle glaucoma using electroretinogram techniques.^63^^,^^64^ The reduced ES with longer dark adaptation suggests that compensatory mechanisms, such as rod or ipRGC-mediated contributions, which appear to be more resistant to the glaucomatous changes in the early stage, may partially mask the deficit, making the impairment less detectable. This observation aligns with the studies reporting that ipRGCs are less susceptible in early glaucoma.^65^^,^^66^ Future studies should consider standardizing dark adaptation duration to optimize the sensitivity of PLR-based assessments for glaucoma.

In addition, our results indicated that studies using longer stimuli (>1 second) are more likely to detect alterations in the amplitude of pupil constriction compared with those using shorter stimuli (≤1 second) (Table 3). This suggests that extended stimulus durations enhance the ability to detect glaucomatous changes in PLR, likely by engaging sustained ipRGC responses. Shorter stimuli may primarily capture the transient phase of PLR, which is thought to be driven by photoreceptors.^23^

This review has several limitations that should be considered when interpreting the findings. First, the included studies exhibited considerable methodological variability, particularly in pupillometry techniques, stimulus characteristics, and dark-adaptation protocols, all of which contributed to the substantial heterogeneity observed across outcomes. In addition, none of the studies reported participants’ light-adaptation status prior to undergoing dark adaptation, potentially influencing PLR measurements and limiting comparability between studies. Moreover, several PLR metrics were evaluated in only a small number of eligible studies, reducing the precision of pooled effect estimates for these parameters. These limitations highlight the need for future studies employing standardized testing protocols, consistent reporting practices, and individual-level data analyses to improve the reliability and interpretability of PLR metrics in glaucoma research.

## Conclusion

Although this meta-analysis suggests that amplitude of constriction is the most affected PLR metric and identifies stimulus conditions that may enhance sensitivity, these findings should be interpreted with caution.

## Declaration of Generative AI and AI-Assisted Technologies in the Writing Process

During the preparation of this work, the author(s) used Copilot to improve readability and language. After using this tool/service, the author(s) reviewed and edited the content as needed and take(s) full responsibility for the content of the publication.
