# Supplementary material for: Assessing Pupil Light Reflex Metrics in Glaucoma: Insights from a Systematic Review and Meta-Analysis
Source: Ophthalmol Sci. 2026 May 14;6(7):101225. doi: 10.1016/j.xops.2026.101225 (PMC13284455; doi:10.1016/j.xops.2026.101225)
Supplement: Table S1 [file mmc1.pdf]

Table S1. Search strategy used in the PubMed databases

1	“Reflex, Pupillary”[Mesh]
2	(pupil light reflex*[Title/Abstract]) OR (pupil light response*[Title/Abstract])) OR (pupillary response*[Title/Abstract])) OR (pupillography*[Title/Abstract])) OR (pupillometry*[Title/Abstract])
3	"Glaucoma"[Mesh]
4	Glaucoma*[Title/Abstract]
5	#1 OR #2
6	#3 OR #4
7	#5 AND #6
