# Supplementary material for: Assessing Pupil Light Reflex Metrics in Glaucoma: Insights from a Systematic Review and Meta-Analysis
Source: Ophthalmol Sci. 2026 May 14;6(7):101225. doi: 10.1016/j.xops.2026.101225 (PMC13284455; doi:10.1016/j.xops.2026.101225)
Supplement: Table S2 [file mmc2.pdf]

Table S2. Inclusion and exclusion criteria

Inclusion Criteria	Exclusion Criteria
Articles published in English language	Studies that were published before 2000
Studies that evaluated glaucoma and normal groups	Studies which present data as between eye or within eye difference
Studies with well-explained methodology	Studies with non-extractable pupil data
