# Supplementary material for: Assessing Pupil Light Reflex Metrics in Glaucoma: Insights from a Systematic Review and Meta-Analysis
Source: Ophthalmol Sci. 2026 May 14;6(7):101225. doi: 10.1016/j.xops.2026.101225 (PMC13284455; doi:10.1016/j.xops.2026.101225)
Supplement: Table S3 [file mmc3.pdf]

Table S3. Risk of bias assessment

study	Were the criteria for inclusion in the sample clearly defined?	Were the study subjects and the setting described in detail?	Was the exposure measured in a valid and reliable way?	Were objective, standard criteria used for measurement of the condition?	Were confounding factors identified?	Were strategies to deal with confounding factors stated?	Were the outcomes measured in a valid and reliable way?	Was appropriate statistical analysis used?
Chang et al.2013	Yes	Yes	Yes	Yes	Yes	Yes	Yes	Yes
Quan et al.2023	Yes	Yes	Yes	Yes	Yes	Yes	Yes	Yes
Ahmadi et al. 2020	Yes	Yes	Yes	Yes	Yes	Yes	Yes	Yes
Bayraktar et al. 2023	Yes	Yes	Yes	Yes	Yes	Yes	Yes	Yes
Feigle et al. 22011	Yes	Yes	Yes	Yes	Yes	Yes	Yes	Yes
Duque-Chique et al. 2018	Yes	Yes	Yes	Yes	Yes	Yes	Yes	Yes
Gracitelli et al. 2014	Yes	Yes	Yes	Yes	Yes	Yes	Yes	Yes
Gracitelli et al. 2015	Yes	Yes	Yes	Yes	Yes	Yes	Yes	Yes
Kankipati et al. 2010	Yes	Yes	Yes	Yes	Yes	Yes	Yes	Yes
Kelbsch et al. 2016	Yes	Yes	Yes	Yes	Yes	Yes	Yes	Yes
Martucci et al. 2014	Yes	Yes	Yes	Yes	Yes	Yes	Yes	Yes
Najjar et al. 2018	Yes	Yes	Yes	Yes	Yes	Yes	Yes	Yes
Najjar et al. 2021	Yes	Yes	Yes	Yes	Yes	Yes	Yes	Yes
Adhikari et al. 2016	Yes	Yes	Yes	Yes	Yes	Yes	Yes	Yes
Tatham et al. 2013	Yes	Yes	Yes	Yes	Yes	Yes	Yes	Yes
Tatham et al. 2014	Yes	Yes	Yes	Yes	Yes	Yes	Yes	Yes
Quan et al. 2023	yes	yes	yes	yes	yes	yes	yes	yes
Wride et al. 2009	Yes	Yes	Yes	Yes	No	No	Yes	Yes
Nissen et al. 2014	Yes	Yes	Not clear	Yes	Yes	Yes	Yes	Yes
Pillai et al. 2019	Yes	Yes	Yes	Yes	Yes	Yes	Yes	Yes
Chen et al. 2008	Yes	Yes	Yes	Yes	Yes	Yes	Yes	Yes
Carle et al. 2015	Yes	Yes	Yes	Yes	Yes	Yes	Yes	Yes
Pradhan et al. 2017	Yes	Yes	Yes	Yes	Yes	Yes	Yes	Yes
