# Supplementary material for: Assessing Pupil Light Reflex Metrics in Glaucoma: Insights from a Systematic Review and Meta-Analysis
Source: Ophthalmol Sci. 2026 May 14;6(7):101225. doi: 10.1016/j.xops.2026.101225 (PMC13284455; doi:10.1016/j.xops.2026.101225)
Supplement: Table S4 [file mmc4.pdf]

Table S4. Comparison of effect sizes in mild and sever glaucoma. PIPR = post-illumination pupil response.

PLR metrics	Mild glaucoma Hedge's g (95% CI)	Severe glaucoma Hedge's g (95% CI)
**Amplitude of constriction**	-0.72 (-0.83 to -0.60)	-1.10 (-1.20 to -1.00)
**Duration of constriction**	0.35 (0.20 to 0.50)	-0.20 (-0.47 to 0.07)
**Duration of Dilation**	0.43 (0.18 to 0.67)	0.97 (0.50 to 1.45)
**Latency of constriction**	0.70 (0.49 to 0.91)	NA
**Velocity of constriction**	-0.49 (-0.59 to -0.39)	-0.87 (-1.14 to -0.59)
**Velocity of dilation**	-0.26 (-0.38 to -0.13)	-0.55 (-0.88 to -0.23)
**PIPR**	-0.68 (-0.97 to -0.40)	-0.66 (-1.36 to 0.03)
