# Supplementary material for: Assessing Pupil Light Reflex Metrics in Glaucoma: Insights from a Systematic Review and Meta-Analysis
Source: Ophthalmol Sci. 2026 May 14;6(7):101225. doi: 10.1016/j.xops.2026.101225 (PMC13284455; doi:10.1016/j.xops.2026.101225)
Supplement: Figure S1 [file mmc5.pdf]

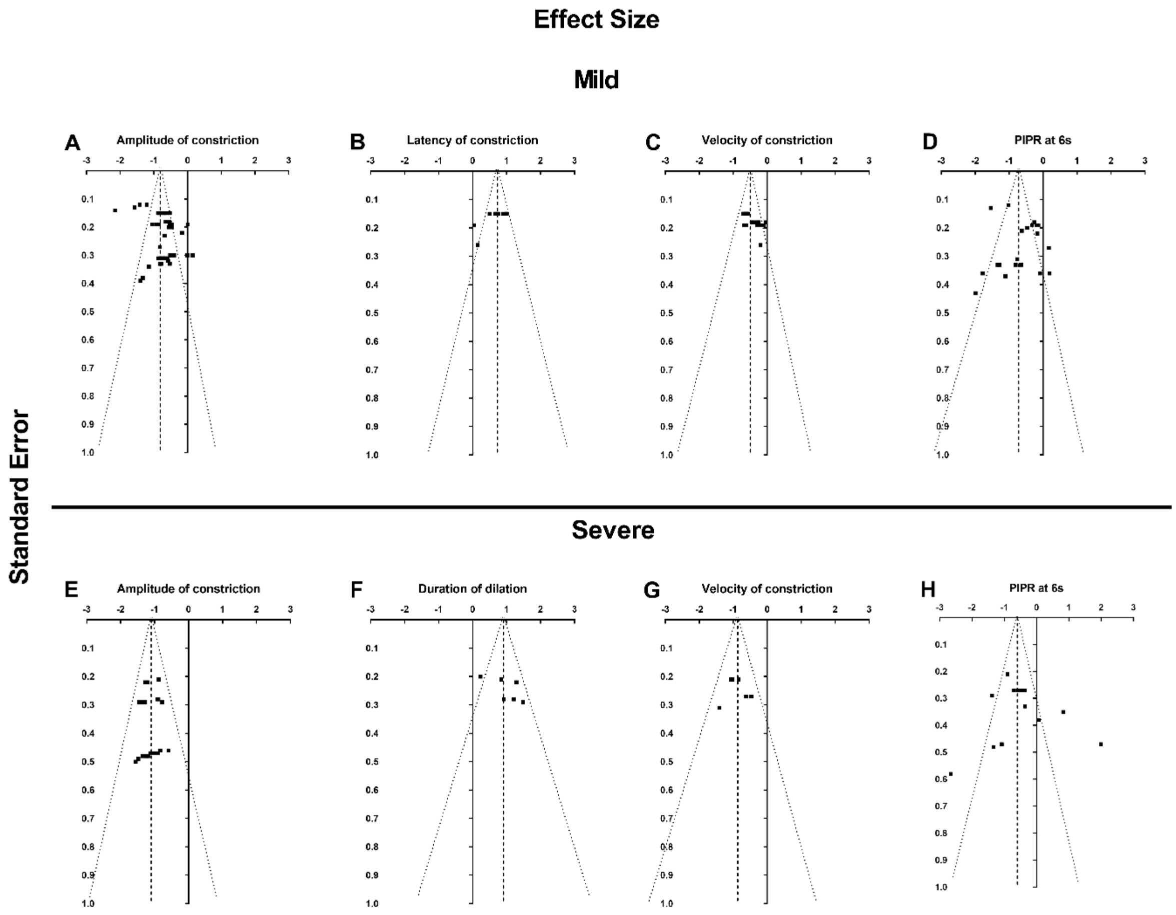

Figure S1: Funnel plots assessing publication bias across pupillometric outcome measures, presented separately for mild (A–D) and severe (E–H) glaucoma groups. Each panel displays the distribution of individual study effect sizes plotted against their standard errors.
