# Supplementary material for: Assessing Pupil Light Reflex Metrics in Glaucoma: Insights from a Systematic Review and Meta-Analysis
Source: Ophthalmol Sci. 2026 May 14;6(7):101225. doi: 10.1016/j.xops.2026.101225 (PMC13284455; doi:10.1016/j.xops.2026.101225)
Supplement: Figure S5 [file mmc9.pdf]

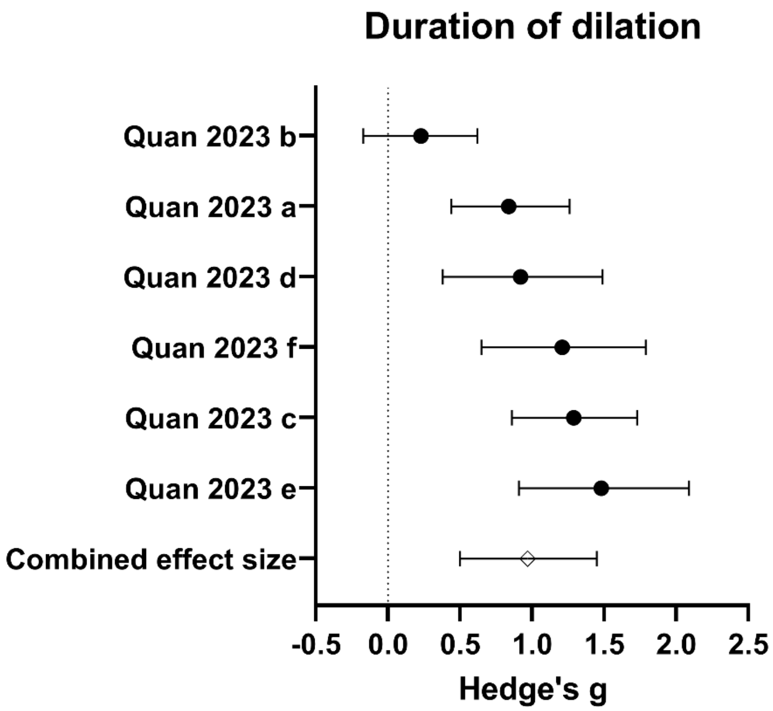

Figure S5. Forest plot of effect sizes for studies evaluating the duration of dilation in severe glaucoma. Data are presented in Hedge's g and 95% confidence intervals.
